# Usefulness of the Nipple Delay Procedure in Nipple-Sparing Mastectomy

**DOI:** 10.3390/jcm15020426

**Published:** 2026-01-06

**Authors:** Koshi Matsui, Emi Kanaya, Shiho Nagasawa, Misato Araki, Shinichi Sekine, Mutsuki Furukawa, Ameri Urasaki, Toshihiko Satake, Tsutomu Fujii

**Affiliations:** 1 Department of Surgery and Science, Faculty of Medicine, Academic Assembly, University of Toyama, Toyama 930-0194, Japan; kmatsui@med.u-toyama.ac.jp (K.M.); emik423@med.u-toyama.ac.jp (E.K.); nagasawa@med.u-toyama.ac.jp (S.N.); arammi33@med.u-toyama.ac.jp (M.A.); sekky@med.u-toyama.ac.jp (S.S.); mutsu555@med.u-toyama.ac.jp (M.F.); s2250017.utm@gmail.com (A.U.); 2 Department of Plastic and Reconstructive Surgery, Faculty of Medicine, Academic Assembly, University of Toyama, Toyama 930-0194, Japan; toshi@med.u-toyama.ac.jp

**Keywords:** nipple-sparing mastectomy, nipple–areolar complex, Nipple Delay, breast reconstruction, ischemic complication, NAC necrosis

## Abstract

**Background/Objectives**: Nipple-sparing mastectomy (NSM) is a surgical procedure that significantly improves postoperative cosmetic outcomes and quality of life (QOL) while ensuring oncological safety. However, ischemic necrosis of the nipple–areolar complex (NAC), resulting from impaired blood flow, remains a serious complication, particularly in patients with risk factors. To mitigate this ischemic risk, the Nipple Delay (ND) procedure, which applies the principle of surgical delay, has been proposed. The objective of this study was to retrospectively review cases in which the ND procedure was performed prior to NSM with immediate autologous breast reconstruction and to evaluate the safety and clinical utility of this technique in preventing NAC necrosis. **Methods**: This study included 30 breasts from 30 patients who underwent the ND procedure prior to NSM with autologous reconstruction at our institution. ND was performed under local anesthesia two weeks before NSM. The skin around the NAC was dissected from the underlying breast tissue. **Results**: The median age of the patients was 49 years, and the mean BMI was 22.7 kg/m^2^. Risk factors for NAC necrosis included periareolar incision in 24 patients (80.0%), a BMI of 25 kg/m^2^ or higher in 7 patients (23.3%), and a history of smoking in 8 patients (26.7%). No cases of full-thickness necrosis requiring NAC excision were observed (0%). Partial-thickness necrosis, which healed with conservative treatment, was observed in 6 patients (20.0%). No malignant involvement was detected in subareolar specimens. **Conclusions**: A staged approach using the ND procedure before NSM suggests effectiveness for preventing serious ischemic complications and safely expanding the indications for NSM, even in patients at high risk of NAC necrosis.

## 1. Introduction

Recent trends in breast cancer surgery have shifted from solely pursuing curative treatment to emphasizing the preservation of postoperative cosmetic appearance and the improvement of patient QOL. Given this trend, NSM, which preserves the NAC, has been widely adopted as a cosmetically oriented procedure. Maintaining the natural form of the breast contributes to patients’ psychological satisfaction, body image, and sexuality, thereby significantly improving QOL [1,2]. Several long-term follow-up studies have established that NSM is an oncologically safe procedure, with local recurrence rates, disease-free survival, and overall survival rates comparable to those of nonnipple-sparing techniques [3,4]. A meta-analysis involving 5594 patients reported a local recurrence rate in the NAC of only 1.3%, widely supporting its oncological safety [5,6,7].

The blood supply to the NAC is complex, primarily derived from the internal mammary artery, lateral thoracic artery, and intercostal arteries. In conventional mastectomy, these deep perforators are sacrificed. Therefore, in NSM, the survival of the NAC relies entirely on the precarious subdermal plexus. Particularly in patients with large breasts or ptosis, the distance from the chest wall to the nipple is long, making this plexus insufficient to maintain adequate perfusion pressure at the distal end of the nipple. This anatomical vulnerability underscores the necessity of a procedure to reinforce the random pattern flap blood supply prior to the main surgery. Specifically, the rapid reduction in blood flow post-mastectomy leads to relative ischemia. While healthy tissues can tolerate this to some extent, the additional stress in high-risk patients—such as those with compromised microcirculation due to smoking or obesity—often exceeds the threshold for tissue survival. Therefore, a strategic intervention to “pre-condition” the vascular network is essential. The risk of postoperative complications is significantly increased in patients with risk factors such as (1) obesity (BMI ≥ 25 kg/m^2^), (2) large breasts (glandular weight ≥ 750 g), (3) significant breast ptosis, (4) a history of smoking, and (5) a history of radiation therapy [8,9].

Furthermore, a periareolar incision as a surgical technique is associated with a very high risk of NAC necrosis [10,11]. To overcome this challenge, the ND procedure, which applies the principle of “surgical delay” long used in plastic surgery, has been proposed [12,13,14,15,16,17]. This is a two-stage procedure aimed at intentionally altering the circulatory environment around the NAC prior to the main NSM. This enhances blood flow in the subdermal plexus, thereby increasing the survival rate of the NAC. Surgical ischemic stimulus is believed to trigger the release of vascular endothelial growth factor (VEGF) and other cytokines, inducing angiogenesis and enhancing blood flow to the NAC [18,19,20].

In recent years, various techniques to improve the blood supply to the NAC during NSM have been reported; however, most of these studies have focused on implant-based reconstruction, and evidence specific to autologous reconstruction remains limited. Because flap thickness and vascular dynamics differ in autologous tissue reconstruction, the risk of NAC ischemia is not necessarily equivalent to that observed in implant-based procedures. In autologous reconstruction, the transferred flap brings well-vascularized, warm tissue to the mastectomy defect, which may theoretically support the ischemic skin flap from underneath. However, this “parasitic” revascularization takes time. In the critical immediate postoperative period, the NAC is vulnerable to ischemia regardless of the reconstruction type, underscoring the need for the delay procedure. Therefore, evaluating the effectiveness of the ND procedure in the setting of autologous reconstruction carries significant clinical relevance.

The present study is characterized by its assessment of the safety and efficacy of ND specifically in the unique context of NSM combined with autologous breast reconstruction. In this study, we retrospectively analyzed our experience with the ND procedure in patients considered at high risk of NAC necrosis who underwent NSM and immediate autologous breast reconstruction to evaluate the safety and clinical utility of the technique.

## 2. Materials and Methods

### 2.1. Patients

This study included 30 breasts from 30 patients who underwent the ND procedure prior to NSM and single-stage autologous breast reconstruction for breast cancer at our institution starting in November 2023. The indication for the ND procedure was not defined by a strict protocol but was based on the surgeon’s assessment of high-risk features for NAC necrosis, such as high BMI, smoking history, severe breast ptosis, or the necessity of a periareolar incision.

### 2.2. Surgical Procedure

**Figure 1:**
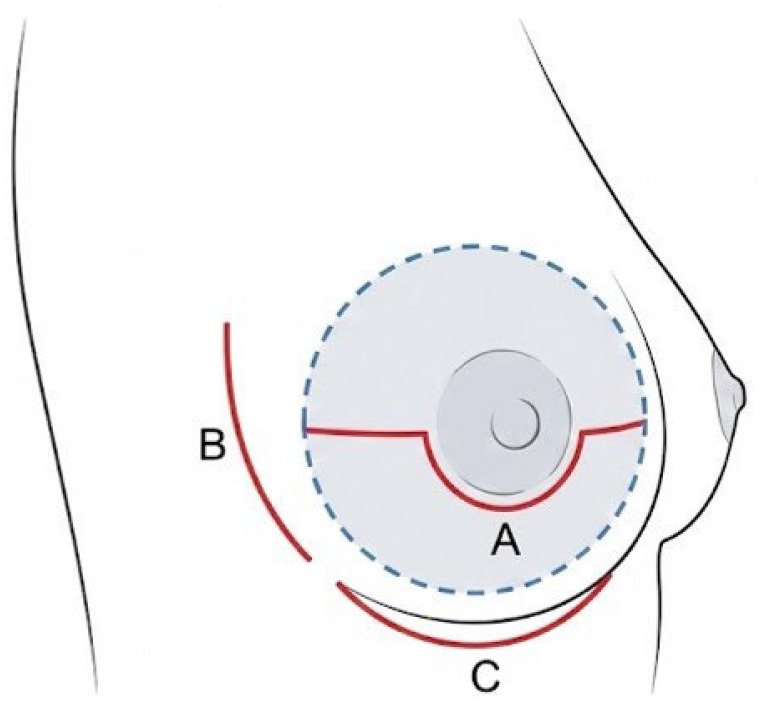
Incision patterns and extent of dissection for the ND procedure. Red solid lines indicate skin incisions: (A) Modified periareolar incision; (B) Lateral incision; (C) Inframammary fold (IMF) incision. The dashed blue line and shaded gray area indicate the extent of subcutaneous undermining beneath the nipple–areolar complex.

ND Procedure: The ND was performed under local anesthesia two weeks prior to NSM and reconstruction, which were conducted under general anesthesia. An incision was made in either a periareolar or lateral mammary location. Through this incision, the skin was dissected from the underlying breast tissue in a radius of at least 5 cm (diameter of at least 10 cm) centered on the NAC (Figure 1).

To minimize thermal injury to the skin flap, hydrodissection was performed using epinephrine saline solution (diluted 1:200,000 epinephrine in normal saline). Approximately 20–30 mL of the solution was infiltrated into the subcutaneous plane beneath the NAC and the surrounding skin. This technique not only minimizes thermal injury but also facilitates the dissection of the correct plane between the subcutaneous fat and the mammary gland, ensuring uniform flap thickness. After the dissection was complete, a small, cone-shaped sample of breast tissue directly beneath the nipple was excised and submitted for permanent histopathological examination. After hemostasis was confirmed, an absorbable hemostatic agent was placed in the subcutaneous space, and the skin was closed without the use of a drain.

NSM and Breast Reconstruction: Two weeks after the ND procedure, the patient underwent surgery under general anesthesia. Approaching through the same incision line as the initial surgery, a total mastectomy was performed. Subsequently, an autologous free flap, harvested from the abdomen or gluteal region, was transferred to reconstruct the breast.

### 2.3. Evaluation Parameters

Based on medical records, the following parameters were retrospectively investigated:

Patient Demographics: Age, BMI, smoking history, comorbidities, and history of neoadjuvant therapy.

Surgical Data: Operative time for NSM and reconstruction, blood loss, and weight of the excised breast tissue.

Histopathological Findings: Presence of malignancy in the subareolar tissue specimen taken during the ND procedure.

Postoperative Complications: Presence of NAC necrosis, skin incision margin necrosis, flap necrosis, infection, thrombosis/embolism, and reoperation.

## 3. Results

### 3.1. Patient Demographics and Surgical Data

Patient demographics and surgical data for the 30 patients are shown in Table 1. The median age was 49 years (range, 36–72 years), and the mean BMI was 22.7 kg/m^2^ (range, 18.0–30.7 kg/m^2^). Risk factors for NAC necrosis included a BMI of 25 kg/m^2^ or higher in 7 patients (23.3%), a history of smoking (current or former) in 8 patients (26.7%), hypertension in 4 patients (13.3%), and diabetes in 1 patient (3.3%). Neoadjuvant chemotherapy was administered to 5 patients (16.7%), and neoadjuvant radiation therapy was administered to 2 patients (6.7%). A periareolar incision was used in 24 patients (80.0%).

The mean operative time for the ND procedure performed under local anesthesia was 40.2 min (range, 32–53 min). The mean operative time for NSM and reconstruction was 559.9 min (range, 345–1005 min), and the mean resected breast weight was 351 g (range, 136–745 g). Pathological examination of the subareolar tissue taken during the ND procedure revealed no cancer cell infiltration in any of the 30 patients.

### 3.2. Postoperative Complications

The incidence of postoperative complications is shown in Table 2. Regarding NAC necrosis, the primary endpoint of this study, there were no cases of severe, full-thickness necrosis requiring NAC excision (0%). Partial-thickness necrosis, characterized by mild superficial necrosis of the NAC, was observed in 6 cases (20.0%). Among the six patients who developed partial necrosis, a stratified analysis of risk factors revealed that two patients had a BMI ≥ 25, and all 6 cases involved a periareolar incision. All of these cases healed promptly with conservative treatment, such as ointment application, without compromising the cosmetic outcome. Other complications included skin incision margin necrosis in 7 cases (23.3%) and infection in 3 cases (10.0%). Partial necrosis of the reconstructed flap occurred in 1 case (3.3%), and reoperation for infection or hematoma removal was performed in 2 cases (6.7%). Complications related to the ND procedure itself included hematomas in 2 cases, both of which resolved conservatively.

## 4. Discussion

This study validated the efficacy of the ND procedure in preventing NAC necrosis, the primary concern in NSM, based on our clinical experience. The results were extremely favorable: full-thickness necrosis requiring NAC excision was completely avoided, even though the study population included patients with known risk factors for NAC necrosis, such as high BMI and a history of smoking. These findings strongly suggest that the ND procedure is highly useful in enhancing the safety of NSM and expanding its indications.

The key to the success of the ND procedure lies in the “surgical delay” concept, which is well established in plastic surgery. By intentionally interrupting a portion of the blood supply, the tissue is exposed to ischemic stress. This stress triggers the release of various growth factors and cytokines, including VEGF, which potently induces the dilation of existing vessels and the formation of a new vascular network (angiogenesis). The two-week interval between the ND procedure and the NSM allows sufficient time for this angiogenic process to proceed, thereby strengthening the subdermal vascular plexus that nourishes the NAC. The techniques employed in our study, such as hydrodissection to avoid thermal injury and the placement of an absorbable hemostatic agent subcutaneously, are also considered important for maximizing this physiological effect.

Meta-analysis reports an overall incidence rate of 4.62% for partial necrosis of the nipple–areolar complex and 2.49% for total necrosis of the nipple–areolar complex. The incidence rate of nipple–areolar complex necrosis was highest in the periareolar incision, at 18.10% [10]. In our study, partial NAC necrosis was observed in six patients (20.0%). However, all of these were minor and confined to the epidermis and healed without compromising the cosmetic result through conservative treatment. This finding demonstrates that, while the ND procedure may not eliminate the risk of necrosis entirely, it plays a decisive role in reducing the severity from “full-thickness” to “partial-thickness” necrosis, ultimately enabling the preservation of the NAC. It is worth noting that our partial necrosis rate of 20.0% appears higher than the 4.62% reported in the meta-analysis. However, this discrepancy is likely due to our strict definition of “necrosis,” which included even minor superficial epidermolysis that healed with simple ointment treatment. In clinical practice, the primary goal of the ND procedure is not merely to prevent minor skin issues but to absolutely avoid full-thickness necrosis that leads to nipple loss. From this perspective, achieving a 0% full-thickness necrosis rate in this high-risk cohort is the most significant indicator of the procedure’s success. Previous studies, such as that by Martinovic et al., who reported favorable outcomes after performing ND in 26 high-risk patients, support our findings [21,22]. Furthermore, the reconstructive modality itself should be considered as a contributing factor to the favorable outcomes. In autologous reconstruction, the NAC is positioned on a well-vascularized flap. Unlike implant-based reconstruction, in which the absence of a biological vascular bed may predispose an ischemic NAC to progression toward full-thickness necrosis, autologous reconstruction allows the underlying transferred tissue to support a compromised NAC, enabling it to survive essentially as a graft. Therefore, the absence of total necrosis observed in our study likely reflects a synergistic effect between the “pre-conditioning” provided by the ND procedure and the “rescue” environment afforded by the autologous tissue flap.

The literature reports several risk factors for NAC necrosis, including the location of the skin incision, smoking status, obesity (BMI ≥ 25 kg/m^2^), and large breasts (glandular weight ≥ 750 g). Our study group included a significant number of patients with these risk factors: 8 patients (26.7%) had a history of smoking, and 7 patients (23.3%) had a BMI of 25 kg/m^2^ or higher. Additionally, the cohort included one patient with a resected breast weight of 745 g, which was near the criteria for macromastia.

An analysis of the relationships between the minor complications observed in this study and these risk factors revealed that of the 6 patients with partial NAC necrosis, 3 (50%) had major risk factors, such as obesity or a history of smoking. Furthermore, in all 6 of these cases, a periareolar incision was used. Traditionally, periareolar incisions are associated with the highest risk of NAC necrosis due to the disruption of the periareolar vascular plexus. Consequently, many surgeons opt for lateral or inframammary fold incisions to prioritize safety, often at the expense of scar visibility. Our study demonstrates that the ND procedure overcomes this trade-off. By surgically delaying the flap, we were able to utilize the periareolar incision in 80% of our cases without a single loss of the NAC. This approach offers the distinct advantage of concealing the scar at the border of the areola, providing a superior aesthetic outcome that aligns with the ultimate goal of NSM. Similarly, of the 7 patients with skin incision margin necrosis, 3 (43%) had risk factors such as obesity or smoking. While this suggests a tendency for complications to occur in patients at high risk of impaired blood flow, it also indicates that even in such high-risk cases, the intervention of the ND procedure limited the degree of necrosis to a partial and conservatively manageable level.

Furthermore, in this study, a subareolar breast biopsy was performed on all patients at the time of the ND procedure, and all results were negative. This can be considered an additional benefit of the ND procedure, as it allows confirmation of oncological safety—a crucial factor in determining suitability for NSM—at a preliminary stage before the final surgery. A similar technique was reported by Jensen et al. [12]. In the event that this biopsy revealed cancer infiltration, the surgical plan could be changed from NSM to a non-nipple-sparing procedure, thus avoiding harm to the patient. Regarding oncologic safety, the two-week interval required for the ND procedure does not lead to a clinically significant delay in definitive treatment. Current guidelines generally allow a window of several weeks between diagnosis (or completion of neoadjuvant therapy) and surgery without compromising survival. Given the profound impact of nipple loss on patient quality of life, we believe the benefit of minimizing severe ischemic complications justifies this brief and oncologically safe interval. Furthermore, receiving confirmation of a cancer-free nipple prior to the major definitive surgery provides significant psychological relief for patients, alleviating anxiety regarding oncological safety.

This study has several limitations. First, it is a single-center, retrospective case series with a limited number of patients (30 cases). Second, as there was no control group that did not undergo the ND procedure, the superiority of the ND method cannot be definitively concluded. Based on the limitations of this study, several avenues for future research are identified. First, prospective, randomized controlled trials are needed to definitively compare NSM outcomes with and without the ND procedure in high-risk patients. Second, quantitative assessment of blood flow is crucial for objective verification. We plan to incorporate intraoperative indocyanine green (ICG) angiography or laser speckle contrast imaging to measure perfusion changes before and after the delay procedure in our future cohorts. Third, from a basic research perspective, analyzing the expression levels of angiogenic factors (e.g., VEGF, HIF-1α) in the tissue sampled during the second surgery could further elucidate the molecular mechanisms underlying the delay phenomenon. These multidimensional approaches will help establish standardized indications and protocols for the ND procedure.

## 5. Conclusions

The staged approach of performing an ND procedure prior to NSM is a feasible and safe strategy. It effectively suppresses the occurrence of severe ischemic complications and enables the preservation of the NAC, even in patients with risk factors for necrosis. We conclude that this method plays a crucial role in safely expanding the indications for NSM and in offering cosmetically superior breast reconstruction to a greater number of patients.

## Figures and Tables

**Table 1:** Patient Characteristics.

Background	*n* = 30
Age (yr), median (range)	49 (36–72)
BMI (kg/m^2^), average (range)	22.7 (18.0–30.7)
BMI ≥ 25	7 (23.7%)
Smoking	8 (26.7%)
Hypertension	4 (13.3%)
Diabetes	1 (3.3%)
Exogenous steroid use	0 (0%)
Neoadjuvant chemotherapy	5 (16.7%)
Preoperative radiotherapy	2 (6.7%)
Skin incision	
Periareolar	24 (80.0%)
Axillary	5 (16.7%)
IMF	1 (3.3%)
Axillary surgery	
SLNB	29 (96.7%)
ALND	1 (3.3%)
Type of reconstruction	
DIEP flap	27 (90.0%)
sGAP flap	3 (10.0%)
Operation time (min), average (range)	559.9 (345–1005)
Breast size (g), average (range)	351 (136–745)

IMF: inframammary fold; SLNB: Sentinel lymph node biopsy; ALND: Axillary lymph node dissection; DIEP flap: Deep Inferior Epigastric Perforator flap; sGAP flap: superior gluteal artery perforator flap.

**Table 2:** Postoperative Results.

Complication	*n* = 30 (%)
NAC necrosis	
Total necrosis	0 (0)
Partial necrosis	6 (20.0)
None	24 (80.0)
Partial necrosis (breast skin)	7 (23.3)
Partial necrosis (free flap)	1 (3.3)
Infection	3 (10.0)
Thrombosis	2 (6.7)
Reoperation	2 (6.7)

## Data Availability

The data presented in this study are available upon reasonable request from the corresponding author. The data are not publicly available due to privacy and ethical restrictions involving patient information.

## Abbreviations

The following abbreviations are used in this manuscript:

NSM	Nipple-Sparing Mastectomy
QOL	Quality of Life
NAC	Nipple–Areolar Complex
ND	Nipple Delay
IMF	Inframammary Fold
SLNB	Sentinel Lymph Node Biopsy
ALND	Axillary Lymph Node Dissection
DIEP	Deep Inferior Epigastric Perforator
sGAP	superior Gluteal Artery Perforator

## References

[1] Didier F., Radice S., Gandini S., Bedolis R., Rotmensz N., Santillo B., Luini A., Galimberti V., Scaffidi E., Lupo F. (2009). Does nipple preservation in mastectomy improve satisfaction with cosmetic results, psychological adjustment, body image and sexuality?. Breast Cancer Res. Treat..

[2] García-Solbas S., Lorenzo-Liñán M.Á., Castro-Luna G. (2021). Long-Term Quality of Life (BREAST-Q) in patients with mastectomy and breast reconstruction. Int. J. Environ. Res. Public Health.

[3] Benediktsson K.P., Perbeck L. (2008). Survival in breast cancer after nipple-sparing subcutaneous mastectomy and immediate reconstruction with implants: A prospective trial with 13 years median follow-up in 216 patients. Eur. J. Surg. Oncol. (EJSO).

[4] Jensen J.A., Orringer J.S., Giuliano A.E. (2011). Nipple-sparing mastectomy in 99 patients with a mean follow-up of 5 years. Ann. Surg. Oncol..

[5] Mota B.S., Riera R., Ricci M.D., Barrett J., de Castina T.B., Atallah A.N., Bevilacqua J.L. (2016). Nipple- and areola-sparing mastectomy for the treatment of breast cancer. Cochrane Database Syst. Rev..

[6] Headon H.L., Kasern A., Mokbel K. (2016). The oncological safety of nipple-sparing mastectomy: A systematic review of the literature with a pooled analysis of 12,358 procedures. Arch. Plast. Surg. (APS).

[7] De La Cruz L., Moody A.M., Tappy E.E., Blankenship S.A., Hecht E.M. (2015). Overall survival, disease-free survival, local recurrence, and nipple-areolar recurrence in the setting of nipple-sparing mastectomy: A meta-analysis and systematic review. Ann. Surg. Oncol..

[8] Spear S.L., Willey S.C., Feldman E.D., Cocilovo C., Sidawy M., Al-Attar A., Hannan C., Seiboth L., Nahabedian M.Y. (2011). Nipple-sparing mastectomy for prophylactic and therapeutic indications. Plast. Reconstr. Surg..

[9] Davies K., Allan L., Roblin P., Ross D., Farhadi J. (2011). Factors affecting post-operative complications following skin sparing mastectomy with immediate reconstruction. Breast.

[10] Daar D.A., Abdou S.A., Rosario L., Rifkin W.J., Santos P.J., Wirth G.A., Lane K.T. (2019). Is There a Preferred Incision Location for Nipple-Sparing Mastectomy? A Systematic Review and Meta-Analysis. Plast. Reconstr. Surg..

[11] Park S., Yoon C., Bae S.J., Cha C., Kim D., Lee J., Ahn S.G., Roh T.S., Kim Y.S., Jeong J. (2020). Comparison of complications according to incision types in nipple-sparing mastectomy and immediate reconstruction. Breast.

[12] Jensen J.A., Lin J.H., Kapoor N., Giuliano A.E. (2012). Surgical delay of the nipple-areolar complex: A powerful technique to maximize nipple viability following nipple-sparing mastectomy. Ann. Surg. Oncol..

[13] Martinez C.A., Reis S.M., Boutros S.G. (2016). The nipple-areola preserving mastectomy: The value of adding a delay procedure. Plast. Reconstr. Surg. Glob. Open.

[14] Bertoni D.M., Nguyen D., Rochlin D., Hernandes-Boussard T., Meyer S., Choy N., Gurtner G.C., Wapnir I.L. (2016). Protecting nipple perfusion by devascularization and surgical delay in patients at risk for ischemic complications during nipple-sparing mastectomies. Ann. Surg. Oncol..

[15] Meli E.Z., Cattin F., Curcio A., Manna E., Samorani D., Tognali D., Gennaro M., Loreti A., Folli S., Fortunato L. (2019). Surgical delay may extend the indications for nipple-sparing mastectomy: A multicentric study. Eur. J. Surg. Oncol..

[16] Karian L.S., Therattil P.J., Wey P.D., Nini K.T. (2017). Delay techniques for nipple-sparing mastectomy: A systematic review. J. Plast. Reconstr. Aesthet. Surg..

[17] Lee P.L., Ma I.T., Schusterman M.A., Beiriger J., Ahrendt G., Cruz C., Diego E.J., Steiman J.G., McAuliffe P.F., Gimbel M.L. (2023). Surgical Nipple Delay and its Expanded Indications for Nipple-sparing Mastectomy. Plast. Reconstr. Surg. Glob. Open.

[18] Holzbach T., Neshkova I., Vlaskou D., Konerding M.A., Gansbacher B., Biemer E., Giunta R.E. (2009). Searching for the right timing of surgical delay: Angiogenesis, vascular endothelial growth factor and perfusion changes in a skin-flap model. J. Plast. Reconstr. Aesthet. Surg..

[19] Lineaweaver W.C., Lei M., Mustain W., Oswald T.M., Cui D., Zhang F. (2004). Vascular endothelium growth factor, surgical delay, and skin flap survival. Ann. Surg..

[20] Jiang Z., Li X., Chen M., Lu L., Gong X. (2019). Effect of Endogenous Vascular Endothelial Growth Factor on Flap Surgical Delay in a Rat Flap Model. Plast. Reconstr. Surg..

[21] Martinovic M.E., Pellicane J.V., Blanchet N.P. (2016). Surgical delay of the nipple-areolar complex in high-risk nipple-sparing mastectomy reconstruction. Plast. Reconstr. Surg. Glob. Open.

[22] Miles O.J., Wiffen J.L., Grinsell D.G. (2022). Nipple delay prior to nipple-sparing mastectomy: The protective effect on nipple-areola complex ischaemia. J. Plast. Reconstr. Aesthet. Surg..

